# FPattNet: A Multi-Scale Feature Fusion Network with Occlusion Awareness for Depth Estimation of Light Field Images

**DOI:** 10.3390/s23177480

**Published:** 2023-08-28

**Authors:** Min Xiao, Chen Lv, Xiaomin Liu

**Affiliations:** School of Physics and Microelectronics, Zhengzhou University, Zhengzhou 450001, China; minxiao@gs.zzu.edu.cn (M.X.);

**Keywords:** light field, depth estimation, deep learning, occlusion handling

## Abstract

A light field camera can capture light information from various directions within a scene, allowing for the reconstruction of the scene. The light field image inherently contains the depth information of the scene, and depth estimations of light field images have become a popular research topic. This paper proposes a depth estimation network of light field images with occlusion awareness. Since light field images contain many views from different viewpoints, identifying the combinations that contribute the most to the depth estimation of the center view is critical to improving the depth estimation accuracy. Current methods typically rely on a fixed set of views, such as vertical, horizontal, and diagonal, which may not be optimal for all scenes. To address this limitation, we propose a novel approach that considers all available views during depth estimation while leveraging an attention mechanism to assign weights to each view dynamically. By inputting all views into the network and employing the attention mechanism, we enable the model to adaptively determine the most informative views for each scene, thus achieving more accurate depth estimation. Furthermore, we introduce a multi-scale feature fusion strategy that amalgamates contextual information and expands the receptive field to enhance the network’s performance in handling challenging scenarios, such as textureless and occluded regions.

## 1. Introduction

With the continuous development of computer vision technology, people are increasingly interested in enhanced 3D technology. In this regard, depth estimation as a fundamental task of 3D vision is of extreme research importance, as it can extract the depth information of a scene from an image. This technique is widely applied in a variety of fields, such as autonomous driving [1], drone navigation [2,3], and 3D reconstruction [4,5], making more intelligent and more effective decisions possible with accurate depth information. In addition, depth estimation can further enhance the user’s 3D experience together with such applications as virtual reality and augmented reality [6,7]. Accurate estimations of depth information are particularly important since depth information is crucial for the interaction and perception capabilities of intelligent systems in various domains. However, when an object in the scene occludes another and only part of the object or surface is visible in the occluded area, it can lead to incomplete information, and thus accurate depth estimation is totally impossible. Yet, the accurate estimation of the relative positions between objects is hard, and occluded scenes would bring difficulty into depth estimation. Solving this problem and improving the depth estimation accuracy of occluded regions will have a positive impact on the overall performance. Therefore, the research on depth estimation for occluded scenes is of great significance.

There are various techniques for depth estimation, including monocular depth estimation [8,9,10] and multi-view stereo matching. Monocular depth estimation involves estimating depth information from a single image or video frame. Due to the absence of geometric constraints from additional viewpoints, monocular depth estimation often faces challenges in accurately inferring depth, especially for distant objects or in scenes with ambiguous texture patterns. Multi-view stereo matching utilizes multiple images captured from different viewpoints for more robust depth estimation. By leveraging disparities between corresponding points in these images, the multi-view stereo method can overcome some of the limitations of monocular depth estimation and provide more accurate depth maps. The depth estimation of light field images can be viewed as a particular multi-view stereo matching method. The unique feature is that the light field image is a type of four-dimensional data set consisting of dense and regular scene samples. This allows for multiple representations of the light-field image, such as epiplane images (EPIs), sub-aperture images (SAIs), and macropixels. This enables the design of more novel methods for the depth estimation of light field images. The light field camera stands out as a distinct camera type capable of collecting and recording light from different directions of a scene. Its unique approach allows users to modify the focal plane or point of view after capturing an image. Modern handheld light field cameras [11] typically employ microlens arrays to capture multi-view images, resulting in dense and regular scene sampling. This characteristic contributes to achieving more accurate depth estimation that traditional multi-view stereo matching methods cannot achieve. One notable advantage of light field cameras is that they are not affected by ambient lighting conditions. This feature makes depth estimation possible even in low-light environments, differentiating light field cameras from conventional ones.

However, light field cameras also possess certain limitations, and the narrow baseline between their sub-aperture images is obviously one of them. This characteristic may bring noise into disparity images, affecting depth estimation accuracy. A trade-off exists between the spatial and angular resolution of light field cameras due to the hardware constraints placed on the image sensor. To address these limitations and achieve accurate depth mapping, Herb et al. [12] proposed a multi-view reconstruction model using kernel parametrization to relax low-rankness, leading to a large-scale convex optimization problem. Jeon et al. [13] utilized the phase shift theorem to estimate sub-aperture image displacement at subpixel levels, introducing a series of methods to improve the accuracy and noise immunity of the cost volume. Zhang et al. [14] explored the application of the geometric structure of 3D lines in light fields for triangulation and stereo matching, presenting a simple yet effective algorithm to enhance depth estimation accuracy. Though these methods show promise, their practicality is uncertain due to the computational complexity. Wanner et al. [15] addressed this issue using local data terms and structural tensor methods for fast depth estimation. However, such fast methods often sacrifice accuracy. In the wake of the development of deep learning, recent proposals have emerged based on convolutional neural networks (CNNs) for depth estimation [16,17]. However, these methods utilize horizontal, vertical, and diagonal views to construct the epi, and while the use of partial views can be effective in reducing computation time, this may not effectively utilize the full information of the light field, hence resulting in a limited improvement in the accuracy of depth estimation.

This paper proposes a new depth estimation network (FPattNet) for light field images. While the full views are input into the network, the attention mechanism is utilized to improve the performance of depth estimation by assigning different weights to each view according to the scene, and the multi-scale feature fusion strategy is utilized to improve the accuracy at the occlusion. Our main contributions are as follows:

1. Occlusion awareness: The depth estimation network is specifically designed to handle occluded scenes. We employ a multi-scale feature fusion strategy that amalgamates contextual information from various levels, enabling the model to handle challenging scenarios, such as textureless and occluded regions.
2. Scene-specific depth estimation: The optimal combination of views for accurate depth estimation can vary from scene to scene. With this view selection module, our network can dynamically adapt its focus to the most relevant information in each scene.

## 2. Related Work

On the basis of light filed images, depth estimation methods can be roughly classified into optimization-based methods and learning-based depth estimation methods. Moreover, since the light field image quality affects the depth estimation results, we briefly review the image quality. Thus, it is possible to undertake better preprocessing based on the image quality.

### 2.1. Quality Assessment of Light Filed Image

Shi et al. [18] introduced the Blind Quality Evaluator of Light Field Images, which utilizes tensor theory to explore the 4D structure characteristics of light field images (LFIs) and proposes Tensor Spatial Characteristic Features for spatial quality and the Tensor Structure Variation Index for angular consistency to assess LFI quality. Shi et al. [19] addressed the challenging problem of Light Field Image Quality Assessment by proposing a No-Reference Light Field Image Quality Assessment scheme. The method quantifies LFI quality degradation by evaluating spatial quality and angular consistency. Zhou et al. [20] proposed a novel tensor-oriented no-reference light field image quality evaluator based on tensor theory. They consider the inherent high-dimensional characteristics of LFI and evaluated quality degradation in both spatial and angular dimensions. Meng et al. [21] proposed a full-reference light field image quality evaluation index that accurately reflects the angular–spatial characteristics of light field data by extracting key refocused images and combining their features with chrominance information. 

### 2.2. Optimization-Based Depth Estimation Methods

Traditional optimization-based depth estimation methods mainly construct depth maps using corresponding algorithms, and then optimize the depth maps using optimization algorithms to improve accuracy, whereas deep learning algorithms benefit from the development of computer hardware and mathematical theory. They only need to input the light field data into the neural network, which can learn the relevant model and perform depth estimation. In contrast, traditional optimization-based methods deal well with problems such as occlusion and noise. However, the drawbacks are that their algorithms are extremely complex and the initial construction is very time-consuming. In addition, when using traditional methods, we need to build different algorithms for different scenes, resulting in less generality in the models compared with deep learning methods. Deep learning methods are efficient and fast with more model generality, but lack specificity in specific scenarios.

Traditional optimization-based depth estimation methods can be divided into three categories: multi-viewpoint stereo matching-based depth estimation methods, EPI-based depth estimation methods, and refocusing-based depth estimation methods.

#### 2.2.1. Methods Based on Multi-View Stereo Matching

The depth estimation method based on multi-view stereo matching evolved from the traditional 2D image stereo constraint. Traditional 2D image stereo matching requires two cameras at a fixed distance to capture the scene simultaneously and obtain a disparity map. The advent of light field cameras makes it possible to acquire disparity from multiple views using a single device, and the camera parameters are fixed. Bishop and Favaro were among the first to estimate depth from multiple views and demonstrated that the method could be implemented on each pixel of a single light field image [22]. Yu et al. investigated geometric relationships in baseline space and introduced an image segmentation algorithm to improve the stereo-matching results [23] further. However, Yu’s method produces significant errors when there is an occlusion in the scene. Park and Lee [24] used communication and scattering cues that were robust to occlusion and noise as constraints, and introduced two data costs: a finite angular entropy cost and a finite adaptive scattering cost. However, these methods do not overcome the problem of the small baselines of light field cameras. Jeon et al. [13] proposed a multi-view cost–volume matching algorithm to evaluate the matching cost, and Heber et al. [12] proposed a new perspective-bending PCA matching condition to improve the sensitivity of the algorithm to subtle pixel-level differences. To further address the occlusion problem, Chen et al. [25] used a superpixel-based regularization method to detect partially occluded regions. They also used confidence maps of labeled regions and edge intensity weights to improve the accuracy of occlusion boundary detection.

#### 2.2.2. Methods Based on EPI

Depth estimation using pairs of polar plane images was first proposed by Bolles et al. [26] in 1987. They used a camera to photograph closely spaced images in a specific scene. They simplified the depth measurement by converting the 3D into 2D through processing steps. Based on this, Matousek et al. [27] proposed a dynamic programming-based algorithm to find the corresponding depth by extracting rows of similar intensity in EPI. In other studies, Criminisi et al. [28] used an EPI volume and a dense horizontal rectification time volume. They generated a set of EPI volumes with the same depth using linear translation. In contrast to the EPI-based approach described above, Wanner [15] and Goldluecke used the dominant direction of the EPI to estimate depth in their structural tensor approach. However, this method is not sensitive to noise and occlusion. In addition, the depth estimation method based on the EPI of 2D images may lead to significant estimation errors due to very dark and bright image features. Tao et al. [29] proposed a dense depth estimation method based on a complete 4D light field EPI, combined with scattered focus and corresponding depth cues, to obtain better depth maps from light field images. They obtained the scatter focus cues by calculating the spatial variance after angular integration. Similarly, Zhang et al. [14] proposed a method that uses a rotating parallelogram operator (SPO) to locate lines in the EPI and calculate their orientations for local depth estimation. This method can further handle occlusion and has better robustness to noise. In addition, Zhang et al. [14] combined multi-directional SPO with edge directions to improve depth estimation near the occlusion boundary, and demonstrated that the optimal EPI direction is parallel to the occlusion boundary.

#### 2.2.3. Refocusing-Based Methods

Light field images contain information about multiple viewpoints in a scene, and objects of different depths correspond to different disparity values in different viewpoint images. Different focusing effects can be obtained by ordering these images by specific methods. Mousier et al. [30] used the relationship between camera calibration parameters and raw data to convert the full-focus images of light field images into depth maps. The full-focus image of the light field image was transformed into a depth map using the relationship. However, since all passive color cameras have low-contrast regions, the solid color background looks the same regardless of the presence of focus, which means that texture is completely lost in the solid color background. The depth estimation method based on refocusing has better results for textures in complex scenes, but has disadvantages such as time-consuming algorithm design and serious computational difficulty.

These traditional methods require extensive optimization calculations for different scenarios, and there is a trade-off between performance and computational efficiency in traditional methods. In this paper, we use a learning approach to represent depth estimation as a supervised learning task using convolutional neural networks while balancing the efficiency and performance of light field depth estimation to achieve a fast and accurate light field depth estimation algorithm.

### 2.3. Learning-Based Depth Estimation Methods

By learning methods, we can quickly obtain depth maps of light field images and network models with better robustness to occlusions and noise. This approach can effectively control the balance between accuracy and computational cost. Heber et al. [31] extended their previous work on EPI in 2017 using 3D operations instead of 2D operations and RGB-EPI-based neural networks for depth estimation. Shin et al. [16] applied multi-stream networks to their convolutional neural network model to address the problem of insufficient data. Pock et al. [32] proposed a new convolutional neural network model that learns the four-dimensional light field and the corresponding four-dimensional depth field and builds end-to-end mapping, and then uses higher-order regularization in subsequent processing to further refine the depth field and obtain accurate depth values. Tsai et al. [33] implemented the cost volume by shifting each sub-aperture image based on stereo matching, and used cost volume for the first time for light field depth estimation. Chen et al. [34] constructed a cost volume using sub-aperture images of four branches, horizontally, vertically, and diagonally, and utilized an attention mechanism to achieve intra-branch and inter-branch feature fusion. Wang et al. [35] proposed a fast cost constructor that realizes the handling of occlusion by dynamically adjusting the pixels of different views. Wang et al. [36] proposed a disentangling mechanism for light fields by rearranging the sub-aperture images of the light field into macro-pixel images, applying the disentangling mechanism to quickly construct a cost volume.

Since the actual receptive field in the deep network is much smaller than the theoretical receptive field [33], the main problem of current learning-based methods is that they do not effectively utilize global contextual information. In this paper, the expansion of the receptive field is achieved using a feature pyramid network that establishes horizontal connections between different levels of the feature pyramid, and fuses low-level features into high-level features to achieve the extraction of global contextual information. In this way, the accuracy of depth estimation is improved.

## 3. Methodology

**Figure 1:**
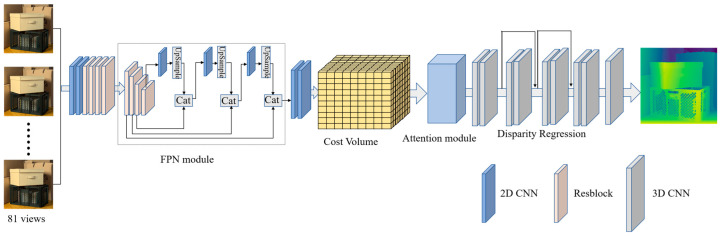
Architecture of the proposed method.

We propose FPattNet, an efficient network designed specifically for light field depth estimation, striking a balance between performance and computational efficiency. The architecture of FPattNet is illustrated in Figure 1, where it takes all sub-aperture images in the light field as input and generates a corresponding disparity map for the central view as output. In the initial stage, the input images undergo feature extraction with two basic convolution blocks, followed by four residual blocks that extract deep features. To expand the receptive field and capture more global information, dilated convolutions [37,38] are applied in the last two residual blocks [39]. Additionally, we incorporate a feature pyramid network [40] to extract contextual information from the input images and construct the cost volume [33] using the feature maps obtained from the feature pyramid output. To reduce redundancy between views and optimize the utilization of available information, we introduce an attention mechanism into the network. This mechanism learns the importance of each view and guides the network to assign different weights to each view, effectively leveraging the sub-aperture images for accurate depth estimation. Finally, we obtain disparity maps by aggregating the cost volume using 3D convolution and performing disparity regression. The detailed structure of FPattNet can be found in Table 1.

### 3.1. Feature Extraction and Feature Pyramid Network

For accurate depth estimation, we leverage the effectiveness of residual blocks for feature extraction. However, the limited receptive field of convolutional neural networks presents challenges in extracting meaningful features, especially in weakly textured or occluded regions. To overcome this limitation, we incorporate a feature pyramid network (FPN) that captures global contextual information by establishing lateral connections between different levels in the feature pyramid, allowing lower-level features to be integrated into higher-level features. This enables the model to comprehend the scene at multiple scales, facilitating more robust depth estimation.

**Figure 2:**
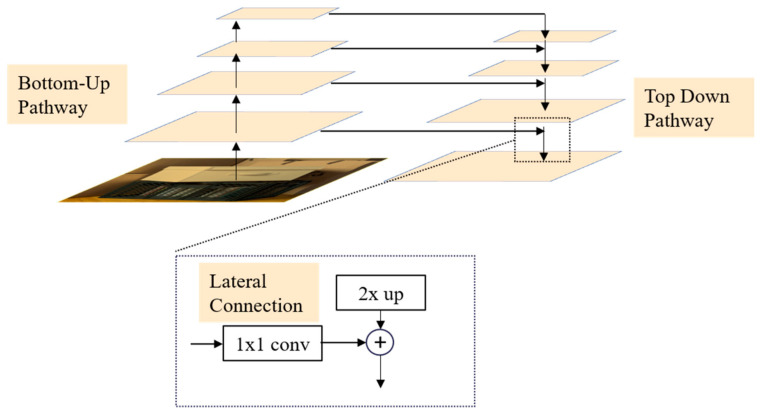
Structure of feature pyramid network.

The structure of the feature pyramid network is depicted in Figure 2. Initially, the FPN module generates four feature maps of varying scales by downsampling the output feature map from the last residual block. These feature maps form a feature pyramid, providing representations at different levels of detail. Subsequently, top-down paths and lateral connections are employed to fuse the feature maps from different levels. This fusion process involves bilinear interpolation for upsampling and 1 × 1 convolutions for dimension adjustment. Ultimately, the features at the bottom of the feature pyramid serve as the output of the FPN module. This hierarchical representation enables a comprehensive understanding of the scene at different scales and captures the relationships between various objects. Consequently, our network achieves more accurate depth estimation, particularly in specialized regions, thereby enhancing overall performance.

### 3.2. Construction Cost Volume

The utilization of a cost volume plays a crucial role in enhancing the accuracy of depth estimation [41,42]. By considering multiple viewpoints of the same scene, the model can effectively explore the disparity space and improve estimation quality. Due to the limited perception boundary of convolutional neural networks and their failure in integrating information from multiple viewpoints, the estimation of disparities with feature maps can lead to inaccuracies, particularly for larger disparities that exceed the network’s receptive field. To overcome this limitation, we construct cost volumes and leverage 3D convolutional neural networks (3DCNN) for cost volume aggregation. This approach allows the model to gather pixel information from larger spatial locations, thereby overcoming the limitation of the receptive field. Consequently, the model can effectively consider pixel correlations across different viewpoints and flexibly explore the disparity space.

In this study, we construct the cost volume by shifting the output feature maps of the feature pyramid across a predetermined range of disparities and concatenating them along the channel dimension. Specifically, given a light field feature F and a candidate disparity $d\in \left\{d_{min},\cdots ,d_{max}\right\}$, we shift the feature map of each view based on the angular coordinates (u, v) of the respective view and the chosen disparity value. Subsequently, we concatenate all the shifted features to construct the cost volume. This process is mathematically described by the following equation:

(1) $$F_{u,v}^{d}(h,w,:)=F_{u,v}\left(h+\left(u_{c}-u\right)d,w+\left(v_{c}-v\right)d,:\right)$$

(2) $$C_{d}=Concat\left(F_{0,0}^{d},\cdots ,F_{U,V}^{d}\right)$$

where $F_{u,v}^{d}$ denotes the shift characteristic of the view (u, v) at disparity *d*, and $C_{d}$ denotes the cost tensor when the disparity is *d*.

The final cost volume is a 4D tensor with the shape of D × H × W × N, where D denotes the range of disparity, H and W are the height and width of the feature map, respectively, and N is the number of channels of the feature map. The disparity range is set to −4 to 4, and 17 levels of disparity are sampled uniformly between them. Finally, accurate subpixel disparity maps are obtained by aggregating cost volumes and disparity regression.

### 3.3. View Selection Module

Light field images, comprising multiple views from different directions, do not contribute equally to accurate depth estimation due to various factors such as occlusions, complex textures, and lighting variations. Moreover, different scenes may require different combinations of views for optimal depth estimation. To overcome these challenges and enhance depth estimation accuracy, we propose a novel attentional view selection module that dynamically adjusts the contribution of views based on scene characteristics and requirements.

Incorporating the Convolutional Block Attention Module (CBAM) [33,43] into our network allows us to integrate both channel attention and spatial attention modules. This integration enables us to adaptively recalibrate the feature response of each view, taking into account its importance in both the channel and spatial domains. By utilizing the CBAM attention mechanism, we can identify and select the most beneficial views for depth estimation, achieving improved accuracy while reducing computational costs.

### 3.4. Disparity Regression and Loss

To obtain aggregated information in the disparity dimension and spatial dimension of the cost volume, we used a 3D convolution-based architecture [41]. Specifically, we constructed a 3D convolutional neural network using eight 3 × 3 × 3 convolutional layers to extract feature information from the custom.

Next, we used soft argmin [44] for disparity estimation. When using cost volume for disparity estimation, the traditional method normally obtains the final disparity map by operating on the cost volume. However, the argument operation is not derivable, and so cannot be trained by backpropagation directly. To solve this problem, we adopt the soft margin method. Unlike the traditional argument, soft is a derivable operator and thus can be optimized through backpropagation during training. Specifically, for each pixel location, we multiply the disparity values in the cost volume with the corresponding normalized probabilities, and weight them to sum and obtain the final disparity values. The predicted disparity calculation formula is defined as:

(3) $$\hat{d}=\sum_{d=-D_{\max}}^{D_{\max}}d\times softmax\left(-c_{d}\right)$$

where the *softmax* is calculated as:

(4) $$softmax\left(x_{i}\right)=\frac{e^{x_{i}}}{\sum_{j=0}^{J}e^{x_{j}}}$$

In light field depth estimation, smoothed *L*_1_ loss is commonly used as a loss function to measure the difference between the predicted depth map and the true depth map. The smoothed *L*_1_ loss function is commonly used, which resists the effects of outliers during training and maintains sensitivity to gradients while reducing overfitting [38]. Disparity loss is defined as:

(5) $$L_{\mathrm{disp}\;}(d,\hat{d})=\frac{1}{N}\sum_{\left(i,j\right)}{\mathrm{smooth}}_{L_{1}}\left(d_{i,j},{\hat{d}}_{i,j}\right)$$

where *N* is the number of predicted labeled pixels, $d_{i,j}$ is the true disparity value, and ${\hat{d}}_{i,j}$ is the predicted disparity value.

(6) $$\mathrm{Smooth}L_{1}(x)=\left\{\begin{array}{rr} 0.5x^{2}, & |x|<1\\ |x|-0.5, & |x|\geq 1 \end{array}\right.$$

## 4. Experiment and Discussion

In this section, we first introduce the dataset used for training, then describe the implementation details, demonstrate the effectiveness of the attention module and the feature pyramid network for improving the performance of depth estimation through ablation experiments, and finally compare with the mainstream methods with qualitative and quantitative results.

### 4.1. 4D Light Field Dataset

This experiment uses the New HCI light field dataset [45], which consists of light field images of 24 scenes. The dataset is divided into four subsets: “additional”, “training”, “test”, and “stratified”. All light field images are generated by the Blender renderer and contain multiple types of scenes, such as occlusion scenes and fine-textured scenes. Each scene has a 9 × 9 light field multi-view sequence with an angular resolution of 9 × 9 and a spatial resolution of 512 × 512 for each light-field photo. In addition, each scene is provided with profiles for camera settings and disparity range, as well as a true depth map.

### 4.2. Implementation Details

In this experiment, we select a subset of the New HCI light field dataset for training and testing. Specifically, we select 16 scenes from this dataset as the training set, where the “additional” subset provides high-quality multi-view light field images, and the “stratified” and “training” subsets provide additional light field images that can be used to validate the network performance. We select four scenes from the “test” subset for the final test. In network training, we randomly select 32 × 32 grayscale blocks as training samples and use data enhancement methods [16] including view shifting, rotation, and scaling techniques. A full 512 × 512 image is used for validation and testing. Our network utilizes the Keras API of the TensorFlow framework and Adam as the optimizer, with the batch size set to 32. Specifically, the first 200,000 iterations take a learning rate of 0.001, and the next 20,000 iterations are fine-tuned with a learning rate of 0.0001. The entire training process was performed on an NVIDIA RTX 3090 GPU, and the training lasts about 100 h.

### 4.3. Evaluation

For an accurate quantitative evaluation of the depth estimation performance, two metrics, BadPix(*ε*) and MSE × 100, were employed, as suggested by prior works [45].

The BadPix metric measures the percentage of pixels for which the absolute disparity value exceeds a specific threshold between the predicted result and the ground truth label. Typically, thresholds of *ε* = 0.01, 0.03, and 0.07 are chosen to evaluate the performance at different levels of precision. A lower BadPix value indicates higher accuracy in the depth estimation algorithm.

The BadPix calculation formula is defined as:

(7) $${BadPix}_{M}(\varepsilon )=\frac{\left|\{x\in M:|d(x)-gt(x)|>\varepsilon \}\right|}{\left|M\right|}$$

The MSE × 100 metric represents the mean square error between the predicted depth map and the ground truth label, multiplied by a factor of 100. This metric quantifies the overall difference between the estimated depth map and the actual depth values. A lower MSE × 100 value indicates a higher level of accuracy in the depth estimation results.

The formula for calculating MSE is:

(8) $${MSE}_{M}=\frac{\sum_{x\in M}\;(d(x)-gt(x){)}^{2}}{\left|M\right|}\times 100$$

A comprehensive quantitative and visual comparison was conducted with twelve state-of-the-art methods. These methods encompassed four traditional approaches [14,24,46,47] and eight deep learning methods [16,33,34,36,48,49]. The results of the BadPix 0.01, BadPix 0.03, BadPix 0.07 and MSE × 100 metrics obtained by these methods across eight scenes from the validation set are presented in Table 2.

**Figure 3:**
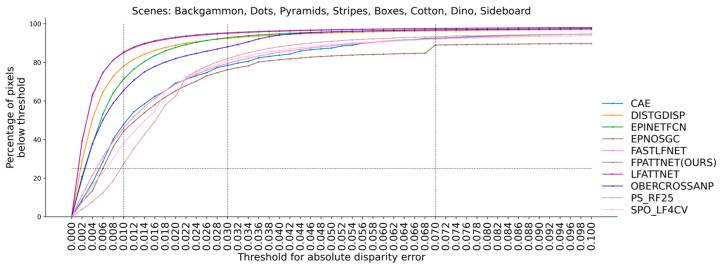
Comparison of our top-ranked methods on the HCI 4D LF benchmark for the percentage of correct pixels at different BadPix thresholds.

As can be seen from Table 2, our method achieves 11 best and 14 s-best results in 32 columns. As can be seen from Table 3, our method achieves the best results in the eight scenes of the validation set for the average BadPix 0.01, BadPix 0.03 and BadPix 0.07. This suggests that the use of the full view as input, in conjunction with the attention mechanism and multi-scale fusion strategy, can improve the overall depth estimation accuracy. To further analyze the BadPix metric, Figure 3 provides a visual representation. The figure illustrates the percentage of pixels with errors below, the different absolute error thresholds for disparity, as well as the percentage of pixels with errors below, a given threshold relative to the total number of pixels.

**Figure 4:**
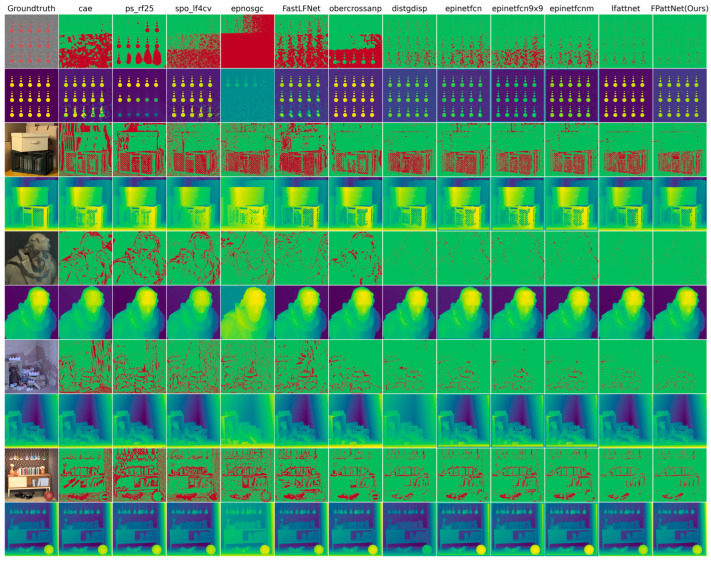
Visual comparison between our method and different LF depth estimation methods on 4D LF benchmarks, including cae [24], ps_rf25 [14], spo_lf4cv [14], epnosgc [49], FastLFNet [48], obercrossanp [47], distgdisp [36], epinetfcn [16], epinetfcn 9 × 9 [16], epinetfcnm [16] and lfattnet [33]. The even-numbered rows show the estimated disparity plots for these methods. In contrast, the odd-numbered rows show the corresponding BadPix 0.03 error maps (pixels with absolute errors greater than 0.03 are marked in red).

The results depicted in Figure 4 indicate that our method consistently outperforms the other methods across various thresholds, particularly at lower thresholds. This observation suggests that our method exhibits higher accuracy in depth estimation compared to the competing approaches.

**Figure 5:**
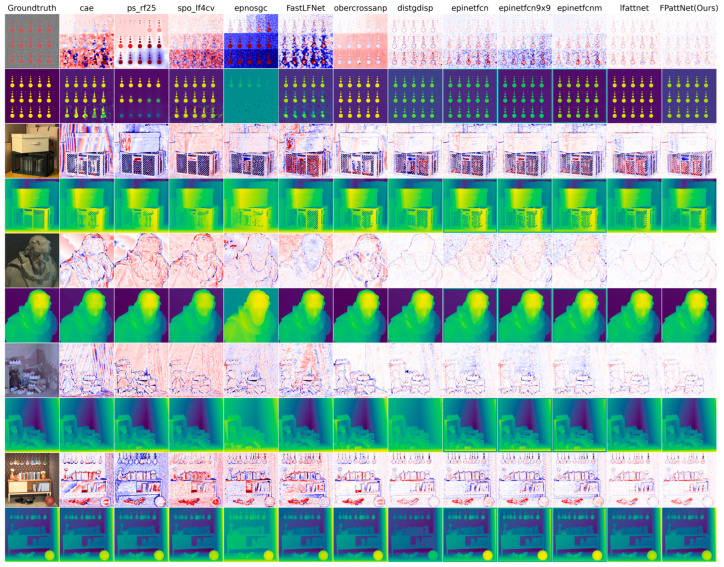
Visual comparison between our method and different LF depth estimation methods on 4D LF benchmarks, including cae [24], ps_rf25 [46], spo_lf4cv [14], epnosgc [49], FastLFNet [48], obercrossanp [47], distgdisp [36], epinetfc [16], epinetfcn 9 × 9 [16], and epinetfcnm [16] and lfattnet [33]. The even-numbered rows show the estimated disparity plots for these methods. In contrast, the odd-numbered rows show the corresponding MSE error maps (pixels that predict the correct disparity are marked white, with red pixels indicating too far and blue pixels indicating too close).

Figure 4 and Figure 5 present visual comparisons of different methods in five distinct scenes: dots, box, dino, sideboard, and book. Each scene represents different challenges in depth estimation, such as coplanar circles with clear edge structures in dots, severe occlusion in box and sideboard, and textureless regions, particularly in the book in the scene box.

By examining the BadPix 0.03 bad pixel map, our method consistently exhibits significantly fewer bad pixels compared with the other methods, particularly in scenes with occlusion and textureless regions. This indicates that our method is more effective in the accurate estimation of depth, even in some challenging scenes. In the scene dots, our method captures circular structures with greater precision and minimizes the number of bad pixels. In scenes with severe occlusion, such as box and sideboard, our method demonstrates excellent performance in handling occluded regions and generates more accurate depth maps. Additionally, our method excels in estimating depth in textureless regions, as evidenced by the book in the scene box. While other methods may struggle to estimate depth accurately in such regions, our method manages to capture the depth information more reliably, resulting in fewer bad pixels.

These visual comparisons further validate the effectiveness of our method in achieving accurate depth estimation, particularly in scenes with occlusion and textureless regions. The reduced number of bad pixels highlights the superior performance and robustness of our approach compared with the competing methods.

**Figure 6:**
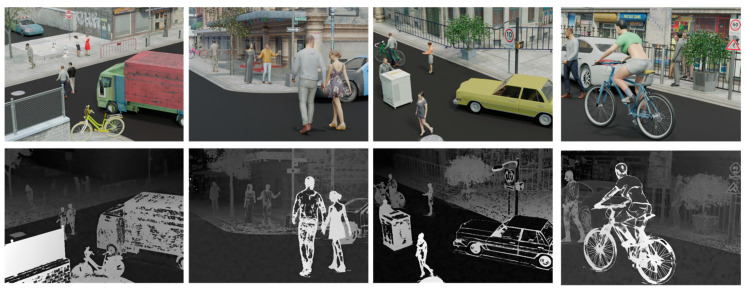
Results of our method for the UrbanLF dataset. The first row represents the image of the center view and the second represents the disparity image.

**Figure 7:**
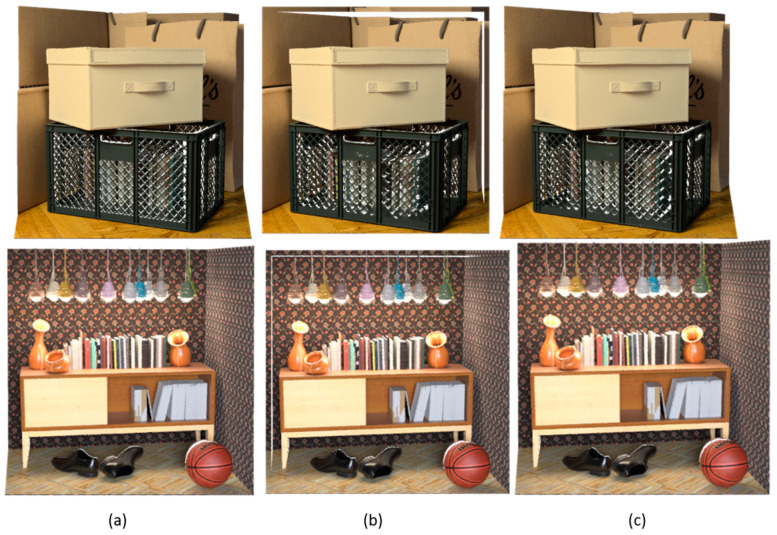
Comparison of 3D point clouds generated by different methods in the scenes boxes and sideboard. (**a**) Ground truth, (**b**) epinetfcn [16], (**c**) FPattNet (ours).

Figure 6 shows the performance of our method applied to the UrbanLF [50] dataset. Figure 7 shows the 3D point cloud reconstructed with the depth map obtained by different methods, and it is clear that the 3D point cloud reconstructed by our method has better integrity compared with epinetfcn, which is close to ground truth.

### 4.4. Ablation Experiment

In this section, we demonstrate the effectiveness of the FPN module and attention module with a large number of ablation experiments. 

#### 4.4.1. The Effect of the Feature Pyramid Networks

To investigate the impacts of the Feature Pyramid Network (FPN) module on the performance of light field depth estimation, an experiment was conducted in which the FPN module was removed from the original model and retrained. The results, as presented in Table 4, demonstrate that the FPattNet model achieved a decrease of 0.521 in average Mean Squared Error (MSE) and a decrease of 3.881 in BadPix values. These findings suggest that incorporating the FPN module can significantly enhance the overall performance of the depth estimation algorithm.

**Figure 8:**
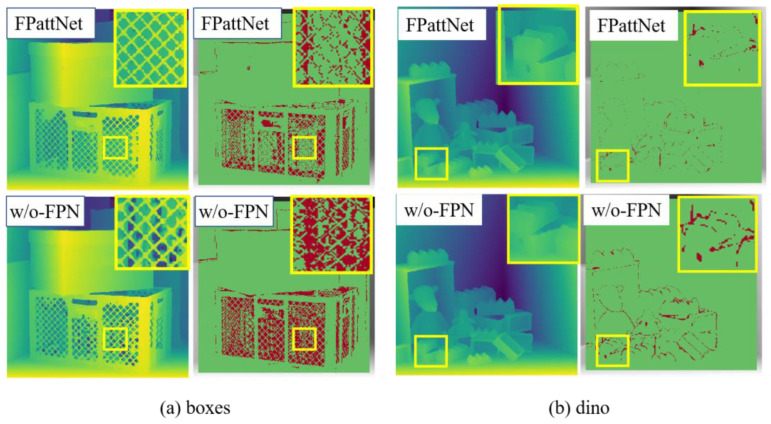
Visual comparison of our method on scenes (**a**) boxes and (**b**) dino with/without FPN. The first column shows the estimated disparity map and the second column shows the BadPix 0.03 error maps.

Additionally, Figure 8 provides a visual comparison of the disparity and bad pixel maps between the models with and without the FPN module in the Boxes and Dino scenes. The figure clearly illustrates that the utilization of the FPN module leads to a substantial improvement in depth estimation accuracy, particularly in heavily occluded regions and textureless regions. This improvement can be attributed to the multi-layer architecture of the FPN module, which facilitates feature extraction at various scales. Consequently, the network can capture rich semantic information and effectively model structures and textures of different sizes. In contrast, traditional single-scale methods often struggle to capture complete depth information in occluded regions. By integrating the FPN module, the network can model occluded regions at multiple scales, compensating for the lack of information in occluded and weakly textured regions, thereby resulting in more precise depth estimates.

#### 4.4.2. The Effect of the View Selection Module

**Figure 9:**
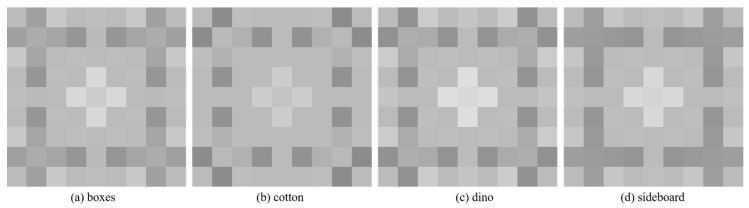
Attention maps in different scenes; the darker the color, the greater the weight.

To validate the effectiveness and superiority of the view selection module, an experiment was conducted to investigate its impact on the performance of light field depth estimation. In this experiment, the Convolutional Block Attention Module (CBAM) was removed from the original model and retrained. The results, as presented in Table 5, demonstrate a notable improvement in the network’s performance when the attention mechanism is applied. The average Mean Squared Error (MSE) and BadPix values in the eight scenarios with the attention mechanism decreased by 0.379 and 4.836, respectively, compared with the variant without the attention mechanism. The introduction of the attention mechanism enables the network to dynamically select and emphasize the most valuable views for depth estimation. As shown in Figure 9, the view weights differ from scene to scene. This is accomplished by dynamic weight assignment to each view based on its respective contribution. Views with higher contributions are assigned higher weights, allowing the network to better utilize the depth information provided. Conversely, views with lower contributions are given lower weights, thereby minimizing their impact on the final depth estimation results. Focusing on the most informative views, this adaptive weighting of views improves the accuracy and robustness of depth estimation.

**Figure 10:**
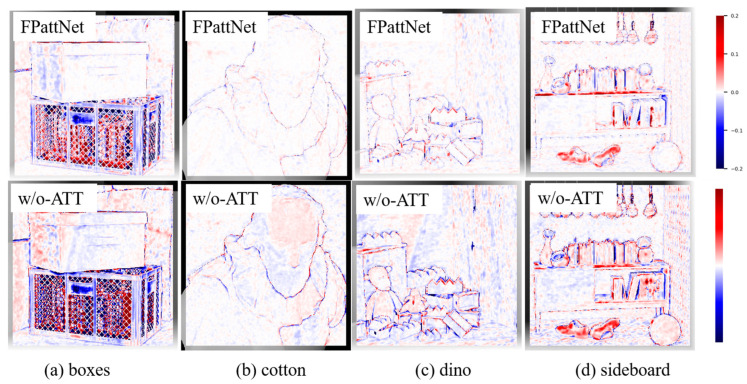
Visual comparison of our method on scenes (**a**) boxes, (**b**) cotton, (**c**) dino, and (**d**) sideboard with/without using the attention mechanism. The first row shows the MSE error maps when the attention mechanism is used, and the second row shows the MSE error maps when the attention mechanism is not used. (Pixels that predict the correct disparity are marked white, with red pixels indicating too far and blue pixels indicating too close).

The qualitative results depicted in Figure 10 visually prove the effectiveness of the attention mechanism in enhancing depth estimation accuracy. The comparison clearly illustrates that the introduction of the attention mechanism significantly improves the accuracy of depth estimation.

## 5. Conclusions

This paper introduces a novel light field depth estimation network that integrates a feature pyramid and an attention mechanism. These additions are designed to enhance the accuracy and robustness of depth estimation, particularly in challenging scenarios. The attention mechanism dynamically adjusts and assigns the weights to each view in the light field image, allowing the network to effectively utilize informative views while minimizing the impact of less reliable ones. This adaptive weighting scheme improves depth estimation accuracy, especially in regions with occlusion and weak texture. Additionally, our feature pyramid module plays a crucial role in capturing contextual information and aggregating features at multiple scales. This multi-scale analysis enables a comprehensive understanding of the scene, leading to improved depth estimation performance. By considering features at different scales, our method effectively captures fine details and accurately estimates depth, even in challenging regions.

Extensive experiments validate the superiority of our method over existing light field depth estimation methods. Our approach outperforms others, generating high-quality depth maps that result in refined and detailed 3D point clouds.

## Figures and Tables

**Table 1:** The detailed structure of our FPattNet. H and W represents the height and width of the input image, respectively. N denotes the number of views and D denotes the number of candidate parallaxes. conv2D, ResBlock2D, Conv3D and ResBlock3D denote the 2D convolution, 2D residual block, 3D convolution and 3D residual block, respectively.

Layers	Kernel Size	Input Size	Output Size
Feature Extraction			
Conv2D_1	3 × 3	N × H × W × 1	N × H × W × 4
Conv2D_2	3 × 3	N × H × W × 4	N × H × W × 4
ResBlock2D × 13	3 × 3	N × H × W × 4	N × (H/16) × (W/16) × 64
FPN Module	1 × 1	N × (H/16) × (W/16) × 64	N × (H/8) × (W/8) × 8
	1 × 1	N × (H/8) × (W/8) × 8	N × (H/4) × (W/4) × 16
	1 × 1	N × (H/4) × (W/4) × 16	N × (H/2) × (W/2) × 24
	1 × 1	N × (H/2) × (W/2) × 24	N × H × W × 32
Cost Volume Construction			
Shift&Concat	-	N × H × W × 4	D × H × W × (4 × N)
Attention Module	1 × 1 × 1	D × H × W × (4 × N)	D × H × W × (4 × N)
Cost Aggregation			
Conv3D_1	3 × 3 × 3	D × H × W × (4 × N)	D × H × W × 150
Conv3D_2	3 × 3 × 3	D × H × W × 150	D × H × W × 150
ResBlock3D × 2	3 × 3 × 3	D × H × W × 150	D × H × W × 150
	3 × 3 × 3		
Conv3D_3	3 × 3 × 3	D × H × W × 150	D × H × W × 150
Cost	3 × 3 × 3	D × H × W × 150	D × H × W × 1
Squeeze&Transpose	-	D × H × W × 1	H × W × D
Disparity Regression			
Softmax	-	H × W × D	H × W × D
Regress	-	H × W × D	H × W × 1

**Table 2:** Quantitative results of comparison with the advanced methods on the HCI_4D LF benchmark. The best results are shown in bold and the second best results are underlined.

	Backgammon				Dots				Pyramids				Stripes			
	0.01	0.03	0.07	MSE	0.01	0.03	0.07	MSE	0.01	0.03	0.07	MSE	0.01	0.03	0.07	MSE
cae [24]	17.32	4.313	3.924	6.074	83.70	42.50	12.40	5.082	27.54	7.162	1.681	0.048	39.95	16.90	**2.405**	3.556
spo_lf4cv [14]	49.94	8.639	3.781	4.587	58.08	35.07	16.27	5.238	79.21	6.263	0.861	0.043	21.88	15.46	14.99	6.955
ps_rf25 [46]	74.66	13.94	7.142	6.892	78.80	17.54	7.975	8.338	83.23	6.235	**0.107**	0.043	41.65	5.790	2.964	1.382
epnosgc [49]	55.98	10.56	3.328	3.699	84.91	82.74	39.25	22.37	28.56	3.169	0.242	0.018	28.17	19.59	18.54	8.731
FastLFNet [48]	39.84	11.41	5.138	3.986	68.15	41.11	21.17	3.407	22.19	2.193	0.620	0.018	63.40	32.59	9.442	0.892
obercrossanp [47]	13.66	4.952	3.413	4.799	73.13	37.66	0.974	1.757	8.171	1.130	0.364	0.008	44.72	9.352	3.065	1.435
distgdisp [36]	26.42	10.72	5.594	4.542	32.05	8.375	2.994	1.525	3.393	0.609	0.188	0.005	17.49	6.836	3.974	0.924
Epinet-fcn [16]	20.90	6.289	3.580	**3.629**	41.05	12.74	3.183	1.635	11.88	0.913	0.192	0.008	15.67	3.115	2.462	0.950
Epinet-fcn 9 × 9 [16]	15.40	4.482	3.287	3.909	44.65	18.71	4.030	1.980	8.913	0.604	0.147	0.007	14.76	2.876	2.413	0.915
Epinet-fcnm [16]	19.44	5.563	3.501	3.705	35.62	9.117	2.490	1.475	11.43	0.874	0.159	0.007	**11.77**	**2.711**	2.457	0.932
AttMLFNet [34]	13.73	4.625	3.228	3.863	**10.61**	2.021	1.606	**1.035**	1.767	**0.429**	0.174	**0.003**	15.44	4.743	2.932	**0.814**
LFattNet [33]	11.58	**3.984**	**3.126**	3.648	15.06	3.012	1.432	1.425	2.063	0.489	0.195	0.004	18.21	5.417	2.933	0.892
FPattNet (Ours)	**11.05**	4.289	3.296	3.808	11.47	**1.540**	**0.960**	1.352	**1.615**	0.436	0.215	0.004	14.07	5.839	3.587	0.838
	boxes				cotton				dino				sideboard			
	0.01	0.03	0.07	MSE	0.01	0.03	0.07	MSE	0.01	0.03	0.07	MSE	0.01	0.03	0.07	MSE
cae [24]	72.69	40.40	17.88	8.424	59.22	15.50	3.369	1.506	61.06	21.30	4.968	0.382	56.92	26.85	9.845	0.876
spo_lf4cv [14]	73.23	29.53	15.89	9.107	69.06	13.71	2.594	1.313	69.88	16.36	2.184	0.310	73.37	28.81	9.297	1.024
ps_rf25 [46]	76.39	35.23	18.95	9.043	70.41	14.98	2.426	1.161	75.97	16.44	4.379	0.751	79.98	36.28	11.75	1.945
epnosgc [49]	67.35	29.01	15.30	9.314	54.85	9.767	2.060	1.406	58.79	12.79	2.877	0.565	66.35	23.87	7.997	1.744
FastLFNet [48]	71.82	37.45	18.70	4.395	49.34	6.785	0.714	0.322	56.24	13.27	2.407	0.189	61.96	21.62	7.032	0.747
obercrossanp [47]	44.96	17.92	10.76	4.750	36.79	7.722	1.018	0.555	22.76	6.161	2.070	0.366	32.79	12.48	5.671	0.941
distgdisp [36]	41.37	20.64	12.83	3.743	6.343	1.216	0.451	0.225	21.68	4.031	1.545	0.132	26.98	8.983	3.768	0.578
Epinet-fcn [16]	49.04	19.76	12.84	6.240	28.07	2.310	0.508	0.191	22.40	3.452	1.286	0.167	41.88	12.08	4.801	0.827
Epinet-fcn 9 × 9 [16]	45.74	18.66	12.25	6.036	25.78	2.217	0.464	0.223	23.45	3.221	1.263	0.151	40.50	11.82	4.783	0.806
Epinet-fcnm [16]	46.09	18.11	12.34	5.968	25.72	2.076	0.447	0.197	19.40	3.105	1.207	0.157	36.50	10.87	4.462	0.798
AttMLFNet [34]	37.66	18.65	11.14	3.842	**1.522**	**0.374**	**0.195**	**0.059**	**4.559**	**1.193**	**0.440**	**0.045**	21.56	6.951	2.691	**0.398**
LFattNet [33]	37.05	18.97	11.04	3.996	3.644	0.697	0.272	0.209	12.22	2.340	0.848	0.093	20.74	7.243	2.870	0.531
FPattNet (Ours)	**33.98**	**16.85**	**9.576**	**3.672**	3.574	0.627	0.197	0.214	9.808	1.975	0.699	0.088	**20.03**	**6.491**	**2.688**	0.466

**Table 3:** Average values of different methods on the validation set. The best results are shown in bold.

Methods	BadPix 0.01	BadPix 0.03	BadPix 0.07	MSE
cae	52.30	21.86	7.743	3.243
ps_rf25	72.63	18.30	6.961	3.694
spo_lf4cv	61.83	19.23	8.233	3.572
epnosgc	55.62	23.94	11.20	5.981
FastLFNet	54.12	20.80	8.153	1.756
obercrossanp	34.62	12.17	3.417	1.823
Distgdisp	21.97	7.677	3.918	1.459
Epinet-fcn	28.86	7.582	3.606	1.705
Epinet-fcn 9 × 9	27.33	7.824	3.579	1.753
Epinet-fcnm	25.75	6.552	3.383	1.655
AttMLFNet	13.35	4.874	2.801	**1.257**
LFattNet	15.07	5.269	2.840	1.350
FPattNet (Ours)	**13.09**	**4.661**	**2.598**	1.304

**Table 4:** BadPix 0.03 and MSE (multiplied by 100) implemented by different variants of our FPattNet on the HCI 4D LF benchmark. “w/o-FPN” means that the feature pyramid module is not used in the model. The best results are shown in bold.

	Backgammon		Dots		Pyramids		Stripes	
	0.03	MSE	0.03	MSE	0.03	MSE	0.03	MSE
w/o-FPN	9.363	4.791	6.641	1.926	0.772	0.007	11.79	0.902
FPattNet	**4.289**	**3.808**	**1.540**	**1.352**	**0.436**	**0.004**	**5.073**	**0.841**
	box		cotton		dino		sideboard	
	0.03	MSE	0.03	MSE	0.03	MSE	0.03	MSE
w/o-FPN	21.87	5.789	1.882	0.401	4.686	0.145	11.32	0.639
FPattNet	**16.85**	**3.672**	**0.627**	**0.197**	**1.975**	**0.088**	**6.491**	**0.466**

**Table 5:** BadPix 0.03 and MSE (multiplied by 100) implemented by different variants of our FPattNet on the HCI 4D LF benchmark. “w/o-ATT” means that the attentional view selection module is not used in the model. The best results are shown in bold.

	Backgammon		Dots		Pyramids		Stripes	
	0.03	MSE	0.03	MSE	0.03	MSE	0.03	MSE
w/o-ATT	11.53	5.019	8.891	2.161	0.855	0.007	8.696	1.109
FPattNet	**4.289**	**3.808**	**1.540**	**1.352**	**0.436**	**0.004**	**5.073**	**0.841**
	box		cotton		dino		sideboard	
	0.03	MSE	0.03	MSE	0.03	MSE	0.03	MSE
w/o-attention	23.04	4.599	1.789	0.255	5.292	0.230	11.53	0.646
FPattNet	**16.85**	**3.672**	**0.627**	**0.197**	**1.975**	**0.088**	**6.491**	**0.466**

## Data Availability

All datasets analyzed in this study are available from the corresponding author upon reasonable request.
